# Arginine methylation of USP9X promotes its interaction with TDRD3 and its anti-apoptotic activities in breast cancer cells

**DOI:** 10.1038/celldisc.2016.48

**Published:** 2017-01-03

**Authors:** Nithya Narayanan, Zhihao Wang, Ling Li, Yanzhong Yang

**Affiliations:** 1 Department of Cancer Genetics and Epigenetics, Beckman Research Institute, City of Hope Cancer Center, Duarte, CA, USA; 2 Division of Hematopoietic Stem Cell and Leukemia Research, Department of Hematology and HCT, Beckman Research Institute, City of Hope Cancer Center, Duarte, CA, USA

**Keywords:** arginine methylation, apoptosis, stress granule, Tudor domain, USP9X

## Abstract

The Tudor domain-containing proteins are characterized by their specific interactions with methylated protein motifs, including methyl-arginines and methyl-lysines. The Tudor domain-containing protein 3 (TDRD3) is one of the major methyl-arginine effector molecules that recognizes methylated arginine residues on histones and the C-terminal domain of RNA polymerase II, and activates transcription. However, majority of the cellular TDRD3 localizes to the cytoplasm and its functions there are still elusive. Here, we have identified ubiquitin-specific protease 9 X-linked (USP9X) as a TDRD3-interacting protein by GST (glutathione *S*-transferase) pull-down and co-immunoprecipitation. Detailed characterization suggests that the interaction between TDRD3 and USP9X is mediated through the Tudor domain of TDRD3 and the arginine methylation of USP9X. This interaction plays a critical role in TDRD3 protein stability, as knockdown of USP9X expression leads to increased TDRD3 ubiquitination. We also found that USP9X co-localizes with TDRD3 in cytoplasmic stress granules and this localization is diminished in Tdrd3-null mouse embryonic fibroblast cells, suggesting that TDRD3 is essential for USP9X stress granule localization. Furthermore, we found that one of the USP9X de-ubiquitination targets, myeloid cell leukemia protein 1, is regulated by TDRD3, indicating that TDRD3 potentially regulates USP9X de-ubiquitinase activity. Finally, we show that knockdown of TDRD3 expression sensitizes breast cancer cells to chemotherapeutic drug-induced apoptosis, likely due to its regulation of USP9X. This study provides a novel candidate strategy for targeting apoptosis pathways in cancer therapy.

## Introduction

Arginine methylation, which is catalyzed by a group of enzymes called protein arginine methyltransferases (PRMTs), is involved in the regulation of various cellular processes both during development and in human diseases [1, 2]. Mechanistically, methylated arginine often provides a recognition motif for specialized protein domains that, in turn, regulate the protein function. The Tudor domain is one of such domains that are characterized by their anti-parallel β-strands that form a barrel-like fold with an aromatic cage to accommodate methylated ligands [3–5]. It has been found in a wide range of eukaryotic systems, including fungi, plants and mammals. The mammalian genome encodes ~30 Tudor domain-containing proteins (TDRDs), which can be divided into classes based on their binding to methyl-arginine or methyl-lysine, and they have important roles in regulating chromatin epigenetics, RNA metabolism, DNA damage response and signal transduction [6–8]. Currently, 15 TDRDs have been characterized as methyl-arginine-binding proteins, most of which are primarily expressed in the germline cells for regulating gametogenesis [6, 9].

TDRD3 is a 744-amino-acid polypeptide with an N-terminal oligonucleotide-binding (OB) fold domain, a ubiquitin-associated (UBA) domain and a C-terminal Tudor domain [10, 11]. It is ubiquitously expressed in all tissues and localized to both the nuclear (30%) and cytosolic (70%) compartments of the cells. In the nucleus, the Tudor domain recognizes two major active methyl-arginine histone marks, H3R17me2a and H4R3me2a, which are deposited by CARM1 (coactivator-associated arginine methyltransferase 1) and PRMT1 (protein arginine methyltransferase 1) [12–14]. It can also interact with the arginine-methylated C-terminal domain (CTD) of RNA polymerase II (RNAP II) [15, 16]. The OB-fold interacts with DNA topoisomerase IIIβ (TOP3B) to resolve negative supercoiled DNA during transcription, providing a mechanism for TDRD3-mediated transcriptional activation [14, 17, 18]. Knockdown of TDRD3 expression in breast cancer cell lines decreases the expression of TDRD3 target genes, including the oncogene c-MYC [14]. In the cytoplasm, the TDRD3–TOP3B protein complex interacts with the Fragile-X syndrome protein FMRP, and is potentially involved in the regulation of mRNA topological stress and translation [10, 11, 18]. In response to cellular stress, TDRD3 accumulates in stress granules (SGs), where it was proposed to function as a translational repressor of key transcripts that are used during the recovery of the cell. Tdrd3^−/−^ embryos generated using gene-trap technology are developmentally normal, and the adults are viable and fertile [14], suggesting that Tdrd3 might not be an essential gene for development. Tdrd3-knockout mouse embryonic fibroblast cells (MEFs) accumulate DNA damage and the B cells from Tdrd3-knockout mice show significantly increased chromosome translocation [14]. In cancer, elevated levels of TDRD3 form part of the gene expression signature that is used to predict an unfavorable prognosis for breast cancer patients [19]. However, whether and how TDRD3 is involved in tumorigenesis are still elusive.

The ubiquitin-specific protein 9 X-linked (USP9X) is a 292-kDa C19 ubiquitin peptidase consisting of a nuclear localization sequence and ubiquitin-like domain in the N terminus and catalytic USP-specific cysteine and histidine motifs in the C terminus [20]. It belongs to a diverse family of USPs, which regulate the stability of a variety of proteins involved in cellular processes such as endocytosis [21], cell adhesion [22] and polarity [23], as well as cell death [24]. Genetic mutations and dysregulations of USP9X expression have been implicated in various human diseases, including neurological disorders and cancers [25, 26]. Intriguingly, both oncogenic and tumor suppressive functions of USP9X have been reported, likely due to genetic background differences across multiple tumor types and stages. For example, USP9X expression is significantly upregulated in human follicular lymphomas and diffuse large B-cell lymphomas, in correlation with increased expression of MCL-1 (myeloid cell leukemia protein), an anti-apoptotic protein [24]. Mechanistically, USP9X interacts with and stabilizes the MCL-1 protein by inhibiting MCL-1 ubiquitination and proteasomal degradation. In patients with multiple myeloma, overexpression of USP9X associates with poor prognosis. Both knockdown and chemical inhibition of USP9X have been shown to efficiently promote cancer cell apoptosis. Conversely, tumor suppressive function of USP9X has been demonstrated in pancreatic ductal adenocarcinomas that contain *KRAS* mutations [27]. Although the molecular mechanisms are still unclear, loss of USP9X expression results in increased tumorigenic transformation and decreased anoikis. Until now, ~40 proteins have been identified as USP9X-interaction proteins and, among them, more than 20 proteins were characterized as USP9X de-ubiquitination substrates. These substrates are involved in a variety of important cellular processes, such as signal transduction (β-catenin [28], epsin [21], SMAD4 [29], SMRUF1 [30]), cell migration and polarity (AF-6 [31], EFA6 [32], MARK4 [33]) and apoptosis (MCL-1 [24], SURVIVIN [34], ASK1 [35]), all of which could contribute to its role in tumorigenesis. However, how USP9X itself is regulated has not been explored.

Here, we have identified a novel interaction partner of USP9X, the methyl-arginine effector molecule TDRD3. Interestingly, this interaction is regulated by arginine methylation of USP9X, which potentially is carried out by PRMT1. USP9X prevents polyubiquitination of TDRD3 in cells. Furthermore, in response to arsenic stress, USP9X localizes to the cytoplasmic SGs, a process that depends on the presence of TDRD3. Knockdown of TDRD3 expression in breast cancer cells decreases the level of MCL-1, a known USP9X substrate, and sensitizes breast cancer cells to chemotherapy drug-induced apoptosis. Therefore, our study identifies TDRD3 as a regulator of USP9X and a potential target for therapeutic induction of apoptosis in breast cancer cells.

## Results

### TDRD3 interacts with the de-ubiquitinase, USP9X

**Figure 1:**
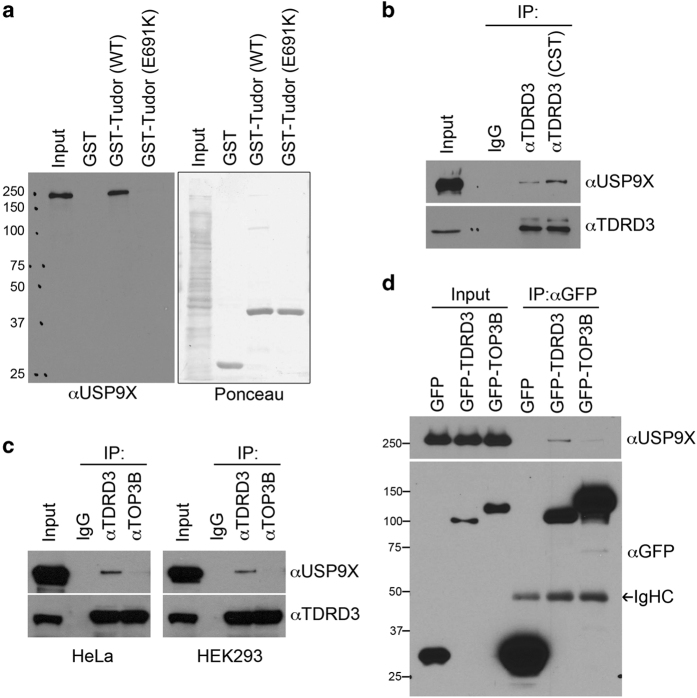
TDRD3 interacts with USP9X. (**a**) GST pull-down assays were performed using recombinant GST, GST-Tudor and GST-Tudor (E691K) proteins with the HeLa cell total cell lysates. Both the input samples and pull-down samples were detected with an anti-USP9X antibody (left panel). The GST-tagged recombinant proteins in the pull-down samples were visualized by Ponceau S staining (right panel). (**b**) TDRD3 and USP9X co-IP. HeLa cells were IPed with rabbit control IgG and two different rabbit polyclonal anti-TDRD3 antibodies. Both the input and the eluted protein samples were detected with anti-TDRD3 and anti-USP9X antibodies. Two different sources of TDRD3 antibody were used to confirm the results—anti-TDRD3 serum [13] and TDRD3 antibody from Cell Signaling Technology (Danvers, MA, USA) (TDRD3 CST). (**c**) TOP3B does not interact with USP9X. Both the HeLa cells and HEK293 cells were IPed with rabbit control IgG, anti-TDRD3 and anti-TOP3B antibodies and detected with anti-TDRD3 and anti-USP9X antibodies. (**d**) HeLa cells transiently transfected with GFP empty vector, GFP-TDRD3 and GFP-TOP3B were IPed with an anti-GFP antibody. The input and IPed protein complexes were detected with anti-GFP and anti-USP9X antibodies.

We previously identified that the Tudor domain of TDRD3 recognizes methyl-arginine motifs on histone tails and activates gene transcription [13, 14]. To further identify TDRD3 interaction proteins, especially the interactions mediated by the Tudor domain, we performed a GST pull-down experiment by incubating HeLa cell lysates with the following recombinant proteins: GST, GST-Tudor domain of TDRD3 (amino acids 588–744) and GST-Tudor domain of TDRD3 (E691K); the TDRD3 E691K mutation has been shown to abolish the interaction between the TDRD3 Tudor domain and methylated arginine motifs [14, 36]. The pull-down samples were subjected to a SDS–PAGE gel followed by Coomassie Blue staining. The protein bands that were visible in pull-down samples from wild-type Tudor, but not Tudor (E691K), were subjected to liquid chromatography-mass spectrometry (LC–MS/MS) for protein identification. As noted before, the TDRD3 interaction proteins are primarily involved in mRNA metabolism and transcriptional regulation, but USP9X was also identified using this approach (data not shown). We further confirmed this result with GST pull-down assays followed by western blotting using a USP9X antibody (Figure 1a). To detect interactions between TDRD3 and USP9X in the cells, we performed co-immunoprecipitation (co-IP) experiments using two different TDRD3 antibodies for IP and as shown in Figure 1b, both TDRD3 antibodies co-IPed USP9X.

TDRD3 tightly associated with TOP3B [14, 17, 18]. To test whether TDRD3 and TOP3B both interact with USP9X, we IPed endogenous TDRD3 and TOP3B from HeLa cells and HEK293 cells and detected their interactions with endogenous USP9X. Surprisingly, endogenous TOP3B did not interact with USP9X, despite its ability to co-IP significant amount of endogenous TDRD3 (Figure 1c). To further confirm that TDRD3, but not TOP3B, interacts with endogenous USP9X, we transiently transfected HeLa cells with GFP empty vector, GFP-TDRD3 or GFP-TOP3B plasmids and IPed with anti-GFP antibody, followed by western blotting with anti-USP9X antibody. Consistent with the endogenous co-IP results, USP9X only interacted with GFP-TDRD3 (Figure 1d). These results demonstrate that USP9X interacts with TDRD3 and suggest that, by interacting with different protein partners, TDRD3 might mediate distinct biological processes.

### USP9X interacts with the C terminus of TDRD3

**Figure 2:**
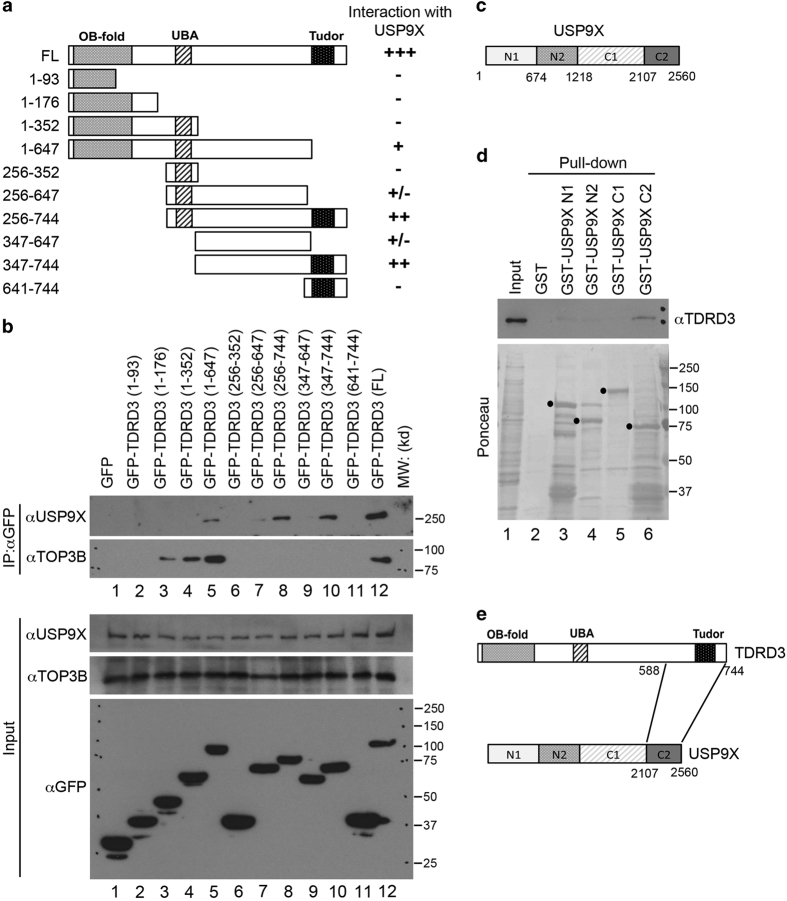
Mapping the interaction regions of TDRD3 with USP9X. (**a**) A series of GFP-fusion deletions of TDRD3 were generated. The locations of the OB fold (OB-fold), the ubiquitin-binding domain (UBA) and the Tudor domain (Tudor) are indicated. A summary of the interactions observed in **b** is shown. (**b**) A co-IP assay was performed in HeLa cells transfected with the different TDRD3 GFP-fusion vectors. The cell lysates were IPed with anti-GFP, and the eluted samples were blotted with anti-USP9X and anti-TOP3B. The input samples were blotted with anti-USP9X, anti-TOP3B and anti-GFP. (**c**) A diagram indicates the GST-fusion deletions of USP9X generated for the pull-down assays described in **d**. (**d**) GST pull-down assays were performed using recombinant GST, GST-USP9X (N1), GST-USP9X (N2), GST-USP9X (C1) and GST-USP9X (C2) with the HeLa cell total cell lysates. Both the input samples and pull-down samples were detected with anti-TDRD3 (upper panel). The GST-tagged recombinant proteins in the pull-down samples were visualized by Ponceau S staining (bottom panel). (**e**) A graphical summary of the protein regions that mediate the TDRD3–USP9X interaction.

Next, we mapped the TDRD3 and USP9X-interaction domains. First, we transiently transfected 11 different fragments of TDRD3 as GFP-fusion proteins into HeLa cells (Figure 2a and b). Immunoprecipitation of this GFP-fusion series revealed that USP9X strongly interacts with the two truncated constructs (256–744 and 347–744) that harbor an intact Tudor domain (Figure 2b, top panel). In addition, the GFP-TDRD3 (1–647) and GFP-TDRD3 (256–647) fragments weakly interact with USP9X. Surprisingly, GFP-TDRD3 (641–744), although contains the Tudor core domain, fails to interact with USP9X. Together with the results from the GST pull-down assay (Figure 1a), which shows the interaction of the TDRD3 extended Tudor domain (588–744) with USP9X, these results suggested that additional structural elements flanking the Tudor core domain structure contribute to its binding property, a common feature also observed in other members of this family [37]. We concluded that the TDRD3 region that interacts with USP9X is located at the C terminus (588–744). To compare the interaction of TDRD3 with USP9X to its interaction with TOP3B, we performed western blotting of the same IPed samples and found that TOP3B interacts with the N terminus of TDRD3 and an intact OB-fold domain is required for this interaction (Figure 2b). Reciprocal mapping experiments were performed for USP9X using GST pull-down experiments, and we found that the C-terminal region of USP9X (2107–2560) is responsible for the TDRD3 interaction (Figure 2c–e). Interestingly, the C terminus of USP9X has been reported to mediate the interactions with its de-ubiquitination substrates, including FOXO3, MIB1 and VMP1 [20], suggesting that USP9X may target TDRD3 for de-ubiquitination; we have tested this hypothesis extensively in the next sections.

### TDRD3 and USP9X interaction is regulated by arginine methylation

**Figure 3:**
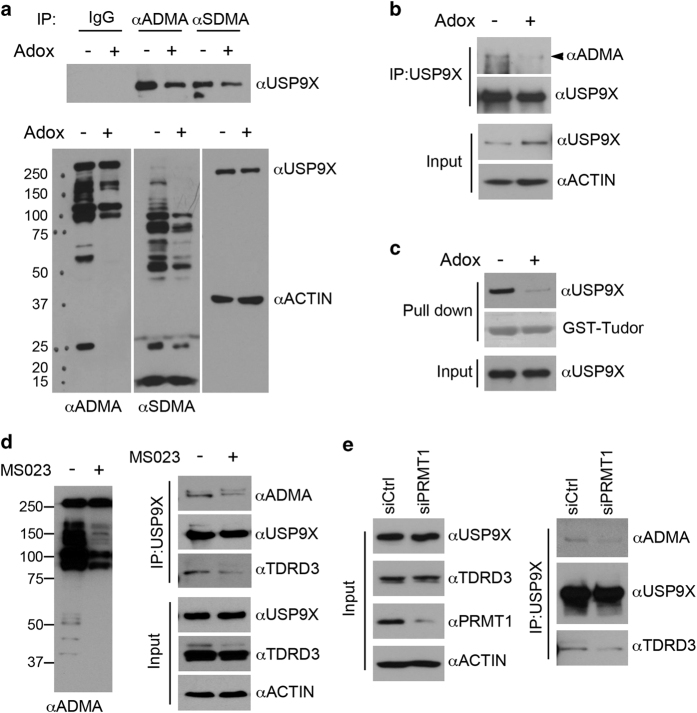
The interaction of TDRD3 with USP9X is regulated by the arginine methylation of USP9X. (**a**) USP9X is arginine-methylated in cells. HeLa cells were first treated with the methylation inhibitor AdOx for 4 days and the total cell lysates were IPed with rabbit control IgG, anti-ADMA (pan-antibody that detects asymmetrically dimethylated proteins) and anti-SDMA (pan-antibody that detects symmetrically dimethylated proteins). The IPed protein complexes were detected with anti-USP9X. The input samples were detected with anti-ADMA and anti-SDMA to monitor the efficiency of the methylation inhibition. Protein expression was detected using anti-USP9X and anti-ACTIN. (**b**) HeLa cells were treated with AdOx for 4 days and the total cell lysates were IPed with anti-USP9X. The eluted protein samples were detected with an anti-ADMA and anti-USP9X. The arrow indicates the USP9X asymmetrical dimethylation. (**c**) The TDRD3 interaction with USP9X is reduced when methylation is inhibited. HeLa cells were treated with AdOx for 4 days to inhibit methylation. GST pull-down assays were performed using recombinant GST-Tudor with HeLa cell lysates. Both the input and the pull-down samples were detected with anti-USP9X. The GST-Tudor proteins in the pull-down samples were visualized by Ponceau S staining. (**d**) ADMA modification regulates USP9X interaction with TDRD3. HeLa cells were treated with PRMT inhibitor MS023 (10μm) for 48 h to inhibit ADMA modification. Co-IP assays were performed to detect USP9X interaction with TDRD3. Both the input and the IPed samples were detected with anti-USP9X, anti-TDRD3 antibody. The effect of MS023 treatment on cellular ADMA modification and USP9X methylation was detected using an anti-ADMA antibody. (**e**) PRMT1 regulates the TDRD3 interaction with USP9X. HeLa cells were transfected with either control or PRMT1-specific siRNA for 3 days and the total cell lysates were IPed with anti-USP9X. The eluted protein samples were detected with anti-TDRD3, anti-ADMA and anti-USP9X antibodies. The expression levels of individual proteins in the input samples were detected with anti-USP9X, anti-TDRD3, anti-PRMT1 and anti-ACTIN.

The Tudor domain of TDRD3 functions as a reader to recognize arginine-methylated protein motifs [6, 36]. The interaction of TDRD3 with USP9X, especially the involvement of the Tudor domain in this process, led us to test whether USP9X is arginine-methylated and whether arginine methylation regulates the TDRD3–USP9X interaction. To test whether USP9X is arginine-methylated in cells, we first treated HeLa cells for four days with adenosine dialdehyde (AdOx), a global methylation inhibitor, and performed IP using pan-asymmetrical dimethyl-arginine (ADMA) and pan-symmetrical dimethyl-arginine (SDMA) antibodies to enrich for arginine-methylated proteins. Compared with the IgG controls, the USP9X proteins were specifically detected in both the pan-ADMA and pan-SDMA antibody-enriched samples (Figure 3a, upper panel). AdOx treatment reduced the detected USP9X signal, suggesting that USP9X is both ADMA- and SDMA-modified. AdOx treatment efficiently reduced both the ADMA and SDMA modifications visible in whole cell extracts (Figure 3a, lower panel, left and middle), but did not change the USP9X protein levels (Figure 3a, lower panel, right). Because the Tudor domain of TDRD3 mainly recognizes ADMA-modified protein motifs [12, 13, 15], we focused on ADMA modification. To further confirm USP9X arginine methylation, we IPed USP9X from control and AdOx-treated HeLa cells and detected its methylation using an ADMA antibody. As shown in Figure 3b, ADMA modification of USP9X was detected and this modification decreased when methylation was inhibited by AdOx (Figure 3b, upper panel). We also performed *in vitro* methylation assays using recombinant C2 domain of USP9X and PRMTs (PRMT1, PRMT3 and CARM1) and confirmed that USP9X is methylated by PRMT1 (Supplementary Figure S1), which is the predominant type I arginine methyltransferase in mammalian cells that accounts for 85% of cellular PRMT activity [38, 39].

To evaluate whether the interaction between TDRD3 and USP9X is regulated by arginine methylation, we performed GST pull-down experiments to compare the interaction of the recombinant TDRD3 Tudor domain with endogenous USP9X from control and AdOx-treated HeLa cells. When methylation was inhibited by AdOx treatment, the Tudor–USP9X interaction was suppressed (Figure 3c), suggesting that USP9X arginine methylation mediates this interaction. We next wished to test whether disruption of ADMA modification can disrupt the TDRD3–USP9X interaction. Therefore, we treated HeLa cells with a recently developed type I arginine methyltransferase inhibitor MS023 (primarily targets PRMT1, PRMT3 and PRMT6) and performed co-IP experiment to detect the TDRD3–USP9X interaction. As expected, MS023 treatment dramatically reduced ADMA modifications of total cellular protein (Figure 3d, left panel). Consistent with this, the level of ADMA-modified USP9X and the extent of TDRD3–USP9X interaction were also reduced, confirming that this interaction is regulated by arginine methylation (Figure 3d, right panel). As USP9X is methylated by PRMT1 *in vitro* (Supplementary Figure S1), we next tested whether PRMT1 is responsible for USP9X arginine methylation and the TDRD3–USP9X interaction in cells. We transfected HeLa cells with control or PRMT1-specific siRNA, and detected the USP9X–TDRD3 interaction by co-IP. Transient knockdown of PRMT1 reduced the USP9X ADMA methylation level and the TDRD3–USP9X interaction (Figure 3e). All together, these results demonstrate that USP9X is arginine-methylated in cells and that the TDRD3–USP9X interaction is regulated by arginine methylation.

### USP9X prevents TDRD3 ubiquitination

**Figure 4:**
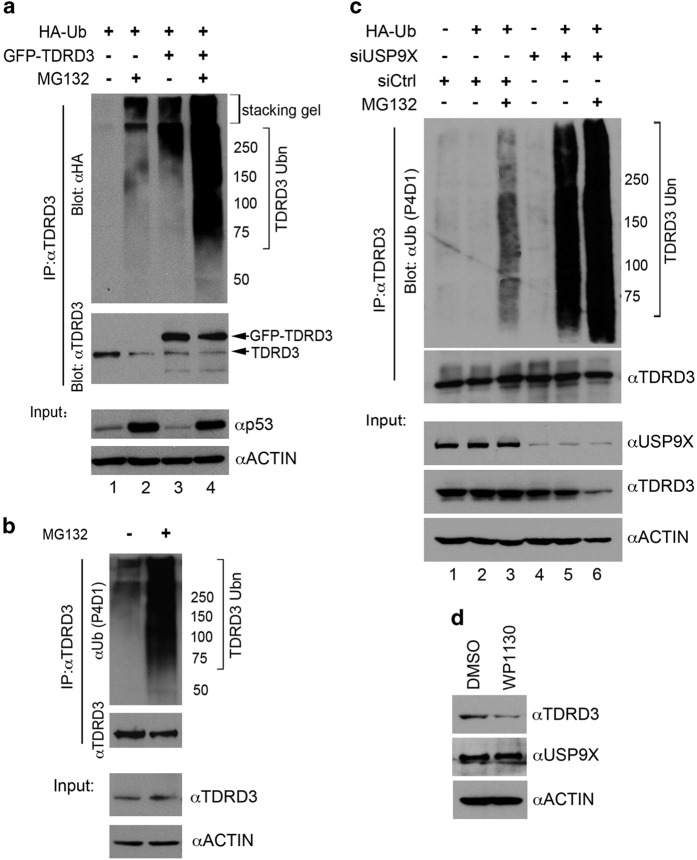
USP9X protects TDRD3 from ubiquitination. (**a**) TDRD3 is ubiquitinated in cells. HeLa cells were transiently transfected with HA-ubiquitin and GFP-TDRD3 (as indicated). After 24 h of transfection, the cells were either treated with DMSO or 10 μm of MG132 for an additional 16 h. TDRD3 *in vivo* ubiquitination was detected by IP with anti-TDRD3. The eluted protein samples were detected with anti-HA and anti-TDRD3. The input samples were detected with anti-p53 and anti-ACTIN to monitor the efficiency of proteasome inhibition by MG132. (**b**) HeLa cells were treated with either DMSO or MG132 for 16 h and the total cell lysates were IPed with anti-TDRD3. The eluted protein samples were detected with anti-ubiquitin (P4D1) and anti-TDRD3. The input samples were detected with anti-TDRD3 and anti-ACTIN. (**c**) Loss of USP9X promotes TDRD3 ubiquitination in the cells. HeLa cells were transfected with either control or USP9X-specific siRNA and either treated with DMSO or MG132 for 16 h. The total cell lysates were IPed with anti-TDRD3 and the eluted protein samples were detected with anti-ubiquitin (P4D1) and anti-TDRD3. The input samples were detected with anti-USP9X, anti-TDRD3 and anti-ACTIN. (**d**) Inhibition of USP9X de-ubiquitinase (DUB) activity destabilizes TDRD3. HeLa cells were treated with 5 μm of WP1130 for 24 h. The expression levels of TDRD3 and USP9X were detected with anti-TDRD3 and anti-USP9X. Anti-ACTIN was used as a loading control.

Because USP9X is a de-ubiquitinase (DUB), we hypothesized that TDRD3 could be a de-ubiquitination substrate of USP9X. First, we tested whether TDRD3 is ubiquitinated in the cells. We performed *in vivo* ubiquitination assays in HeLa cells transfected with GFP-TDRD3 and hemagglutinin (HA)-ubiquitin. When these cells were treated with a proteasome inhibitor (MG132), we observed a dramatic increase in ubiquitination of TDRD3 using an anti-HA antibody (Figure 4a, upper panel, compare lane 1 to lane 2; lane 3 to lane 4). We measured p53 protein levels as a positive control for the efficacy of MG132 treatment and found that p53 was greatly stabilized (Figure 4a, lower panel). We also detected the ubiquitination of endogenous TDRD3 when the HeLa cells were treated with MG132 (Figure 4b). These results demonstrated that TDRD3 is ubiquitinated in cells.

To test whether USP9X de-ubiquitinates TDRD3 in cells, we transfected HeLa cells with either control or USP9X-specific siRNA, and measured TDRD3 ubiquitination. To facilitate the detection of ubiquitination, these cells were treated with either DMSO or MG132. We observed that when the cellular levels of USP9X were reduced by siRNA, the TDRD3 ubiquitination levels markedly increased (Figure 4c, upper panel—compare lane 2 to lane 5) and inhibition of the proteasome greatly augmented this difference (Figure 4c, upper panel—compare lane 3 to lane 6), suggesting that USP9X is necessary to suppress TDRD3 ubiquitination. When evaluating the total cell lysates, we found that the TDRD3 protein levels were reduced in response to USP9X knockdown (Figure 4c, lower panel—compare lanes 1, 2, & 3 with lanes 4, 5, & 6). This result suggests that USP9X has an important role in stabilizing TDRD3 protein levels in cells. To further confirm this and determine whether USP9X DUB activity is required in this process, we applied a pharmaceutical inhibitor of USP9X, WP1130, to HeLa cells and observed that the TDRD3 protein levels decreased in response to WP1130 treatment (Figure 4d). These results demonstrate that TDRD3 is ubiquitinated in cells, and that USP9X stabilizes TDRD3 by preventing its ubiquitination.

### TDRD3 is essential for USP9X stress granule localization

**Figure 5:**
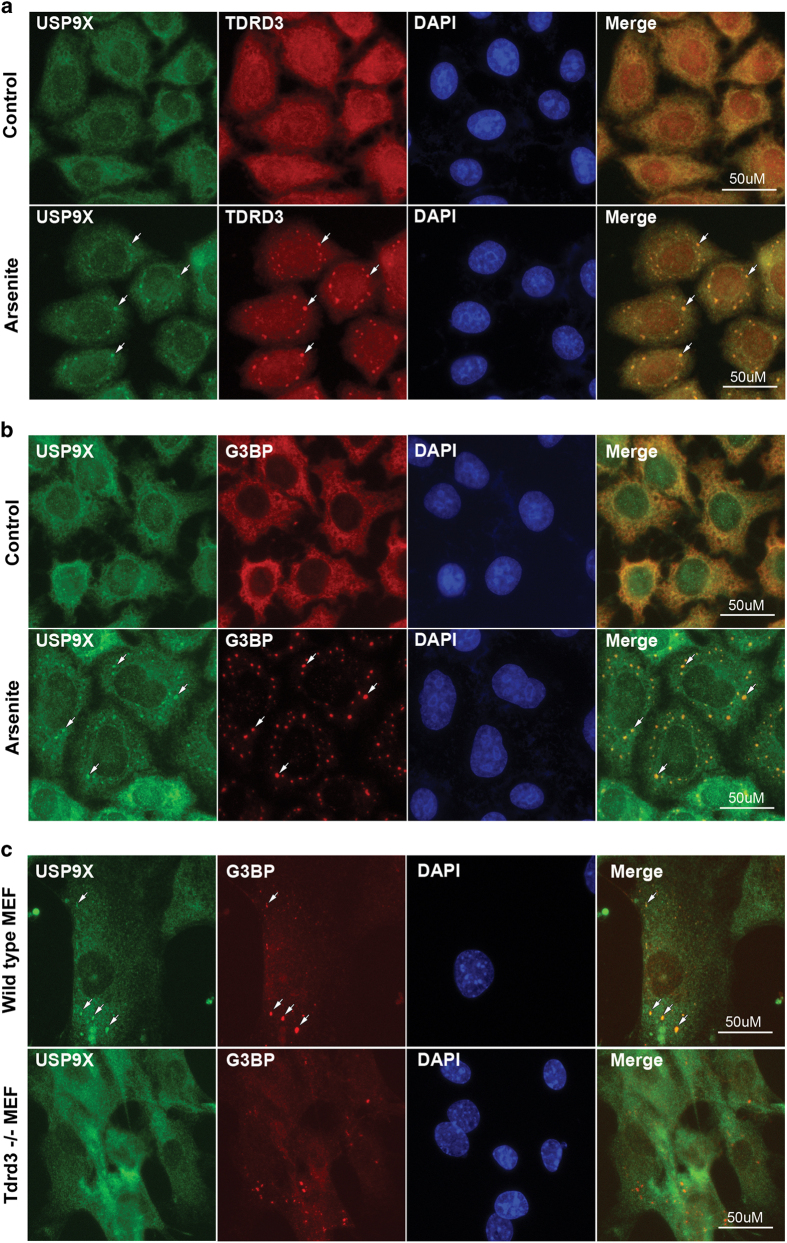
TDRD3 regulates USP9X localization to SGs in response to arsenite treatment. (**a**) HeLa cells cultured on glass coverslips were left untreated (Control) or treated with 0.5 mm sodium arsenite (Arsenite) for 30 min. The cells were fixed and immunostained with anti-USP9X and anti-TDRD3 to detect the localization of endogenous proteins. DAPI was used to stain the nuclear DNA. The arrows indicate USP9X and TDRD3 co-localization in SGs. (**b**) HeLa cells were treated as in **a** and immunostained with anti-USP9X and anti-G3BP. (**c**) Wild-type and TDRD3-knockout MEF cells were both treated with 0.5 mm sodium arsenite for 30 min. The cells were fixed and immunostained with anti-USP9X and anti-G3BP antibodies.

TDRD3 co-localizes with stress granule (SGs) marker proteins, that is, TIA-1-related protein (TIAR) and Ras GTPase-activating protein-binding protein 1 (G3BP), to the cytoplasmic in response to arsenite treatment (Supplementary Figure S2) [10, 11]. The interaction of USP9X with TDRD3 led us to test whether USP9X also localizes to SGs in response to stress. We performed immunostaining assays using HeLa cells that were left untreated or treated with sodium arsenite to induce SG formation. We found that, in the untreated cells, both USP9X and TDRD3 localized mainly to the cytoplasm. However, after arsenite treatment, USP9X localized to the SGs and co-localized with TDRD3 (Figure 5a, white arrows and Supplementary Figure S3A, detected by a different USP9X antibody). To further confirm that the USP9X-immunoreactive granules are indeed SGs, we co-immunostained USP9X with G3BP. In response to arsenite application, USP9X localized to SGs that are positive for G3BP staining (Figure 5b). Transient overexpression of TDRD3 is known to induce SG formation, even without arsenite treatment [10, 11]. To test whether USP9X localizes to SG in response to TDRD3 overexpression, we transiently overexpressed FLAG-TDRD3 constructs in HeLa cells and found that TDRD3 overexpression-induced SG formation in some cells and that USP9X also localized to SGs in these cells (Supplementary Figure S3B). These results demonstrate that USP9X localizes to SGs in response to arsenite-induced stress.

Next, we asked whether USP9X SG localization is regulated by TDRD3. Previously, we have reported the generation of Tdrd3-knockout mice and MEFs [14]. Using these genetically controlled primary cells, we observed that, in arsenite treated wild-type MEFs, USP9X localizes to SGs; this finding is consistent with our observations in HeLa cells (Figure 5c, upper panel). However, USP9X failed to localize to SGs in Tdrd3-null MEF cells, although the SG localization of the marker protein G3BP was not affected (Figure 5c, lower panel). These results suggested that TDRD3 is essential for USP9X SG localization.

### TDRD3 regulates the stability of MCL-1, a USP9X de-ubiquitination target protein

**Figure 6:**
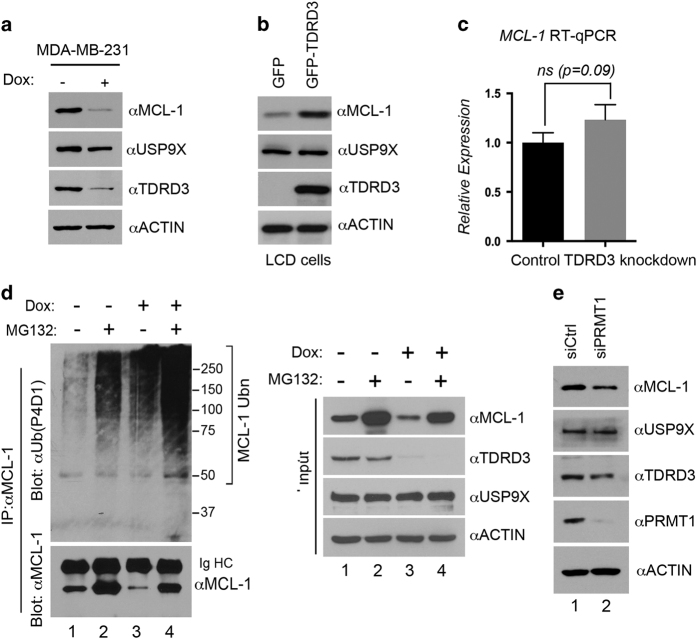
TDRD3 regulates the stability of a USP9X target protein—MCL-1. (**a**) Reduction of TDRD3 levels destabilizes MCL-1. MDA-MB-231 cells were stably transfected with an inducible shRNA vector targeting TDRD3 mRNA. The cells were either left untreated or treated with doxycycline (Dox) (1 μg ml^−1^) for 6 days. The protein expression levels were detected by western blotting using anti-MCL-1, anti-USP9X and anti-TDRD3. Anti-ACTIN served as a loading control. (**b**) The re-expression of TDRD3 stabilizes the MCL-1 protein. LCD cells were stably transfected with GFP or GFP-TDRD3 and the total cell lysates were immunoblotted with anti-MCL-1, anti-USP9X and anti-TDRD3. Anti-ACTIN served as a loading control. (**c**) The mRNA levels of MCL-1 in MDA-MB-231 cells treated using the conditions described in **a** were detected by RT-qPCR (quantitative reverse transcription PCR). Error bars represent the s.d. calculated from triplicate qPCR reactions. (**d**) Reduction of TDRD3 levels promotes MCL-1 ubiquitination. Both control and Dox-inducible TDRD3 shRNA-expressing MDA-MB-231 cells were treated with DMSO or MG132 (10 μm) for 16 h. The cells were lysed in RIPA buffer and IPed with an anti-MCL-1 antibody. The eluted protein samples were detected with anti-ubiquitin (P4D1) and anti-MCL-1 (left panel). The input samples were detected with anti-MCL-1, anti-TDRD3 and anti-USP9X. Anti-ACTIN served as a loading control. (**e**) Reduction of PRMT1 levels destabilizes MCL-1. MDA-MB-231 cells were transfected with control or PRMT1-specific siRNA. The total cell lysates were detected with anti-MCL-1, anti-USP9X, anti-TDRD3 and anti-PRMT1. Anti-ACTIN served as a loading control.

MCL-1 is a member of the anti-apoptotic BCL-2 (B-cell CLL/lymphoma 2) family, which promotes the survival in a variety of cell types [40]. USP9X is known to de-ubiquitinate poly-ubiquitinated MCL-1 and prevent its proteasomal degradation [24]. To further investigate the regulation of USP9X activity by TDRD3, we tested whether TDRD3 regulates MCL-1 protein stability and ubiquitination. Previously, we generated an MDA-MB-231 breast cancer cell line carrying a tetracycline-inducible small hairpin RNA (shRNA) construct to knockdown endogenous TDRD3 levels [14]. When the cells were treated with doxycycline (Dox), we observed an efficient reduction of TDRD3 levels, together with a significant reduction in MCL-1 levels (Figure 6a). In the human VMRC-LCD lung adenocarcinoma cell line, both alleles of TDRD3 are missing [41]. We have previously established a pair of stable cell lines that express GFP empty vector and GFP-TDRD3 [14]. When the MCL-1 protein levels were assayed in these cell lines, we found that re-expression of TDRD3 significantly increased the MCL-1 protein level (Figure 6b). These results suggest that either TDRD3 regulates the expression of MCL-1 or that TDRD3 is required to maintain MCL-1 protein stability. We evaluated the MCL-1 RNA levels after TDRD3 knockdown and detected no significant change in the RNA expression of MCL-1 (Figure 6c), suggesting that the MCL-1 protein is stabilized by TDRD3.

We next evaluated the impact of TDRD3 knockdown on MCL-1 ubiquitination. When MDA-MB-231 cells were treated with MG132, we observed an increase in ubiquitination of MCL-1, as detected with a ubiquitin antibody (Figure 6d, left panel, compare lanes 1 and 2). This laddering is exacerbated when TDRD3 is knocked down in the presence of MG132 (Figure 6d, left panel, compare lanes 3 and 4), even though there is less MCL-1. However, MCL-1 ubiquitination was also enhanced by TDRD3 knockdown in the absence of MG132 (Figure 6d, left panel, compare lanes 1 and 3). This experiment shows that, under normal conditions, MCL-1 is ubiquitinated (at low levels) and that the presence of TDRD3 protects it from enhanced ubiquitination and subsequent targeting to the proteasome for degradation. We did not observe obvious USP9X protein level changes under these conditions (Figure 6d, right panel), suggesting that TDRD3 may be required for USP9X-mediated de-ubiquitination of MCL-1. Indeed, we found that TDRD3 co-IPed with MCL-1 (Supplementary Figure S4A). However, this interaction is not mediated by the Tudor domain of TDRD3 (Supplementary Figure S4B).

Because the TDRD3–USP9X interaction is regulated by PRMT1-mediated arginine methylation (Figure 3), we examined the impact of PRMT1 knockdown on the protein stability of MCL-1. MDA-MB-231 cells were transfected with control or PRMT1-specific siRNA and the protein expression levels were detected by western blotting. We found that MCL-1 protein levels were reduced when PRMT1 was knocked down by siRNA (Figure 6e), confirming that PRMT1 regulates MCL-1 stability, likely through affecting the TDRD3–USP9X interaction. Altogether, these results demonstrated that TDRD3 regulates the ubiquitination and protein stability of MCL-1.

### Knockdown of TDRD3 sensitizes breast cancer MDA-MB-231 cells to apoptosis

**Figure 7:**
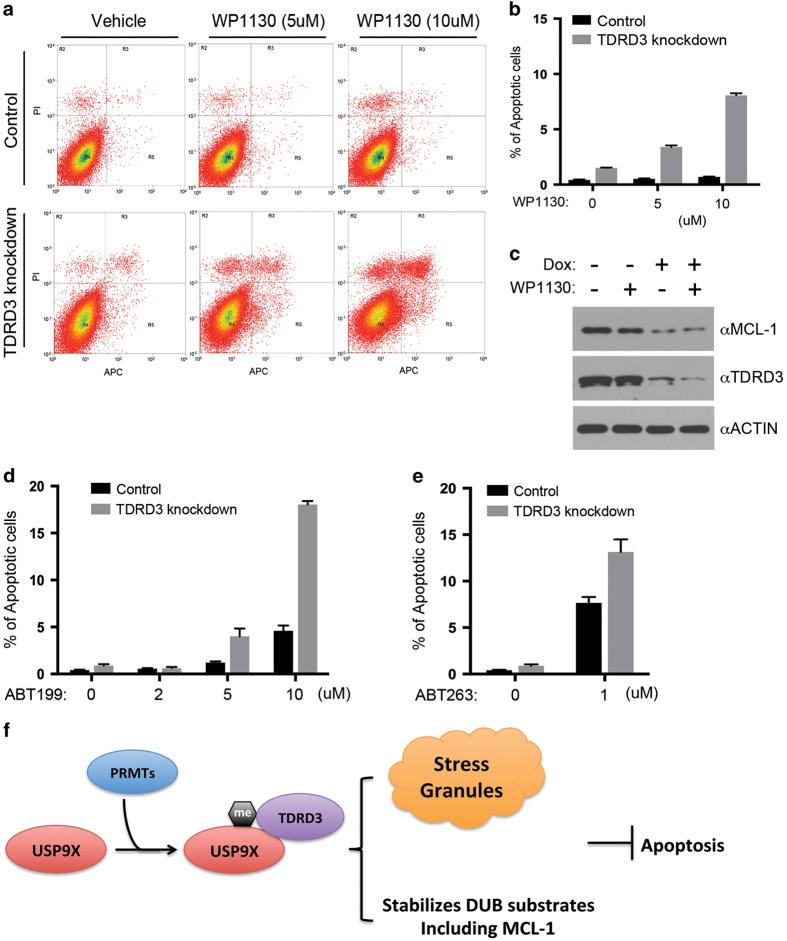
TDRD3 knockdown sensitizes MDA-MB 231 cells to apoptosis. (**a**) Knockdown of TDRD3 sensitizes cells to DUB inhibition-induced apoptosis. Both control and Dox-inducible TDRD3 shRNA-expressing MDA-MB-231 cells were treated with increasing concentration of WP1130 (0, 5, or 10 μm) for 16 h. Apoptosis was detected by FACS analysis. A representative figure is shown. (**b**) Summary of the results from **a** for increasing concentrations of WP1130. (**c**) Western blotting was performed to examine the TDRD3 and MCL-1 protein level in cells treated with or without WP1130 (5 μm, 16 h) and knockdown of TDRD3. (**d**,** e**) Knockdown of TDRD3 sensitizes cells to BCL-2 inhibitor ABT-199 and ABT-263 induced apoptosis. The experiments were performed similar to **a**, except that the cells were treated with increasing concentrations of ABT-199 (0, 2, 5, 10 μm) and ABT-263 (0, 1 μm) for 48 h. Error bars represent s.d. calculated from triplicate assays of one representative experiment. (**f**) Graphic summary of the results. PRMT-mediated arginine methylation of USP9X promotes its interaction with methyl-arginine effector molecule TDRD3, which is essential for its stress granule localization and regulation of its DUB activity, that is, de-ubiquitination of MCL-1. Both processes could contribute to USP9X anti-apoptotic activity.

WP1130 is a partially selective DUB inhibitor that induces apoptosis through accumulation of ubiquitinated proteins. It inhibits USP9X, USP5 and USP14, among others [42]. To assess whether TDRD3 regulates cell viability, we treated control and TDRD3 knockdown MDA-MB 231 cells with increasing concentration of WP1130 and performed fluorescence-activated cell sorting (FACS) analysis. We found that although MDA-MB 231 cells are resistant to WP1130-induced apoptosis within the dose ranges we tested, knockdown of TDRD3 dramatically sensitizes these cells to apoptosis (Figure 7a and b). It was reported that WP1130 treatment reduces MCL-1 level in human lung and colon adenocarcinomas cell lines [43]. To determine if TDRD3 knockdown and WP1130 treatment synergistically reduced MCL-1, we performed western blot and found that either WP1130 treatment or TDRD3 knockdown reduces MCL-1 to different extents (Figure 7c, compare lane 1 and lane 2/3). However, only marginal MCL-1 further decrease was observed in cells with TDRD3 knockdown and WP1130 treatment, compared with TDRD3 knockdown only (Figure 7c, compare lane 4 with lane 3), suggesting that other molecular changes, i.e. altered USP9X SG localization, may be involved in promoting apoptosis.

MCL-1 knockdown only affects the viability of a subset of triple-negative breast cancer cell lines [44]. However, in combination with inhibition of other BCL-2 family members, such as BCL-2 and BCL-2-like protein 1 isoform 1 (BCL-XL), MCL-1 knockdown promotes apoptosis [44]. We then treated control and TDRD3-knockdown MDA-MB-231 cells with two clinically used BCL-2 inhibitor, ABT-199 and ABT-263, and assayed apoptosis. ABT-199 and ABT-263 treatment increased the number of apoptotic cells and knockdown of TDRD3 further enhanced these apoptosis (Figure 7d and e), suggesting that TDRD3 knockdown synergizes with BCL-2 inhibition to induce apoptosis.

We next examined whether knockdown of TDRD3 sensitizes MDA-MB 231 cells to the breast cancer chemotherapy drug, camptothecin (CPT). Control and TDRD3 knockdown cells were treated with increasing amounts of CPT, and apoptosis was assayed by western blot analysis of poly ADP ribose polymerase (PARP) cleavage and FACS analysis. Both assays showed that TDRD3 knockdown sensitizes the cells to CPT-induced apoptosis (Supplementary Figure S5A and B). We also cultured control and TDRD3 knockdown MDA-MB 231 cells with low concentrations of CPT and examined the cell viability after 48 h. Knockdown of TDRD3 significantly reduced the cell viability in the presence of CPT (Supplementary Figure S5C). These results suggest that TDRD3-mediated regulation of USP9X function regulates chemotherapy drug-induced apoptosis, a function that has therapeutic implications for cancer.

## Discussion

We have uncovered a new regulatory pathway for cancer cell apoptosis. This pathway involves the interaction of the methyl-arginine effector molecule, TDRD3, with USP9X. Arginine methylation of USP9X has an important role in this process, as either inhibition of arginine methyltransferase activity or knockdown of PRMT1 expression dampens this interaction. The TDRD3–USP9X interaction is not only important for preventing TDRD3 ubiquitination and degradation, but is also essential for stress-induced localization of USP9X to SGs and USP9X DUB activity towards the anti-apoptotic protein MCL-1, both of which could be the molecular mechanisms that explain the observation that TDRD3 knockdown sensitizes cancer cells to apoptosis (Figure 7f). Because overexpression of TDRD3 has been reported in several human cancers, targeting the TDRD3–USP9X–MCL-1 axis could likely provide a novel therapeutic intervention for cancer treatment.

### TDRD3/TOP3B and TDRD3/USP9X are distinct protein complexes

TDRD3 contains three characterized protein interaction domains, the Tudor domain, the UBA domain, and the OB-fold (Figure 2a). It has been shown to tightly interact with the transcriptional regulator TOP3B, both in the nucleus and the cytoplasm, through its N-terminal OB-fold [14, 17, 18]. On the other hand, the TDRD3 Tudor domain was initially characterized to interact with both ADMA and SDMA [36]. Recent studies from us and others have shown that TDRD3 preferentially recognizes ADMA-modified histones and the C-terminal domain of RNAP II [12, 13, 15, 16], and transduces these signals to facilitate gene transcription. However, other arginine-methylated protein ligands for the TDRD3 Tudor domain have not been identified. In this study, we found that USP9X interacts with TDRD3, but not TOP3B (Figure 1c and d), and that the USP9X–TDRD3 interaction requires the Tudor domain. This finding allowed us to explore novel functions of TDRD3 besides transcriptional regulation.

Multiple studies implicated that USP9X is involved in the establishment of cell-cell tight junctions and cell polarity in epithelial cells [32, 45]. Interestingly, our recently proteomic search of TDRD3 interaction proteins using TAP-tag purification identified a group of proteins involved in cell polarity and migration, including LLGL2, SRGAP2, FMNL2/3, ERC1, PARD3 and ZO-1 [14] (unpublished data). These results indicate that TDRD3 regulates cell polarity and migration. This is in consistent with the clinical observations that elevated TDRD3 level is one of the predictors of poor prognosis for breast cancer patients [19]. It is likely that both nuclear function (as a transcription activator of oncogenes, including c-Myc [14]) and cytoplasmic function (as a regulator of cell polarity and migration) of TDRD3 contribute to breast tumorigenesis.

### USP9X localizes to cytoplasmic stress granules

SGs are cytoplasmic RNA granules composed of stalled translational preinitiation complexes that accumulate under stress conditions, including heat shock, oxidative stress and energy deprivation. SGs are proposed to promote cellular survival pathways, including suppressing stress-responsive MAPK cascades [46] and reducing reactive oxygen species production [47]. Various proteins have been found in SGs, including mRNA binding proteins (PABP1, HuR, G3BP1, SERBP1 and FMRP), translational regulators (TIA-1/TIAR, eIF2a, eIF3 and eIF4), signaling scaffolding proteins (RACK1), histone deacetylase 6 (HDAC6) and TOP3B [17, 18]. All of these SG-associated protein members orchestrate a complex defensive mechanism to protect cells from external insults. In this study, we found that the DUB USP9X localizes to SGs when the cells are stressed with arsenite or transiently overexpress TDRD3 (Figure 5 and Supplementary Figure S3). This dynamic change was diminished in *Tdrd3*-null MEFs, suggesting that TDRD3 mediates USP9X SG localization (Figure 5c). Although the role of USP9X in SGs is still under investigation, another ubiquitin-specific protease (USP10) and its yeast ortholog Ubp3 were found to be essential for efficient SG formation [47, 48], indicating that protein ubiquitination/de-ubiquitination might have a role in the regulation of SG formation. Indeed, de-ubiquitination of TDRD3 could be a major role of USP9X in SGs (Figure 4). Although the direct link between TDRD3 and SG assembly has not been established, stabilization of TDRD3 could help maintain SG integrity, because many SG-associated proteins are arginine-methylated [49]. Furthermore, dysregulation of USP9X has been linked to both human cancers and neurodegenerative diseases [20]. It is worthwhile to examine whether or how USP9X function in SGs regulates the progression of the diseases.

### USP9X interacts with both of the methyl-arginine reader molecules, TDRD3 and SMN

Previously, USP9X was found to interact with another Tudor domain-containing protein, survival motor neuron (SMN) protein [50, 51]. Mutation/deletion of SMN-coding gene, *SMN1*, accounts for 95% of spinal muscular atrophy (SMA), an autosomal recessive disorder that results in degeneration of α-motor neurons in the spinal cord [52]. The interaction of SMN with USP9X prevents its ubiquitination and proteasomal degradation [50]. However, whether and how this interaction is regulated has not been explored. The Tudor domain of SMN was characterized as symmetrical dimethyl-arginine-binding domain [4, 36]. In this study, we found that USP9X is both ADMA and SDMA-modified in cells (Figure 3a). Both the SMN Tudor domain and TDRD3 Tudor domain can pull-down USP9X (Supplementary Figure S6). Furthermore, we showed that the SMN interacting USP9X and the TDRD3 interacting USP9X are from separate pools of total USP9X, because pre-depletion of USP9X with SMN Tudor domain does not affect the abundance of TDRD3 Tudor-interacting USP9X and vice versa (Supplementary Figure S6). These results suggest that ADMA or SDMA modification of USP9X differentially designate its interaction with TDRD3 or SMN, respectively. Future work will be necessary to identify both ADMA and SDMA modification sites on USP9X, and examine how these modifications are regulated by PRMTs to direct USP9X toward different interaction partners for executing diverse cellular functions.

### TDRD3 is a regulator of USP9X function

Although TDRD3 localizes to both the nucleus (30%) and cytoplasm (70%) [10, 11], USP9X mainly localizes to the cytoplasm in most cell types. A large number of proteins have been identified as USP9X de-ubiquitination substrates [20], however, how USP9X itself is regulated has not been revealed until now. In this study, we found that, in addition to being a substrate of USP9X, TDRD3 regulates USP9X DUB activity towards one of its substrates, MCL-1 (Figure 6). Knockdown of TDRD3 expression reduces the MCL-1 protein level, without affecting USP9X expression (Figure 6a and b). Furthermore, TDRD3 co-IPs MCL-1 from cells (Supplementary Figure S4), suggesting that USP9X–TDRD3–MCL-1 can form a stable protein complex. Therefore, it is possible that knockdown of TDRD3 reduces the USP9X–MCL-1 interaction and leads to MCL-1 destabilization. Alternatively, because USP9X failed to localize to SGs when TDRD3 was absent (Figure 5c), it is also reasonable to speculate that USP9X localization to SGs is important for protecting MCL-1 from ubiquitination and degradation. MCL-1 is one of the major pro-survival genes that are expressed at high levels in many types of cancer [40, 53]. Inhibiting USP9X using the small molecule WP1130 triggers tumor cell apoptosis, potentially by decreasing the MCL-1 protein level [42, 43]. Identification of TDRD3 as a regulator of USP9X is important because it provides an alternative pathway to target MCL-1 in cancer. Indeed, knockdown of TDRD3 expression sensitizes breast cancer cells to CPT-induced apoptosis (Supplementary Figure S5).

## Materials and Methods

### Cell lines and antibodies

The human VMRC-LCD cells, LCD cells stably expressing GFP/GFP-TDRD3 and MDA-MB-231 cells expressing Tet-on-sh TDRD3 have been described before [13, 14]. Wild-type and Tdrd3-knockout MEF cells were generated from E12.5 mouse embryos following a standard MEF-generation protocol and the primary MEFs were cultured in Dulbecco’s Modified Eagle Medium (DMEM) containing 10% FBS. The HeLa, HEK293 and MDA-MB-231 cell lines were obtained from ATCC. All of the cell lines were maintained in DMEM containing 10% fetal bovine serum. The anti-TDRD3 antibody has been described before [13]. The anti-TDRD3 (CST) (cat#5942), anti-USP9X (cat#14898), anti-ADMA (cat#13522) and anti-SDMA (cat#13222) antibodies were purchased from Cell Signaling Technology. Anti-MCL-1 (cat# sc-819) was from Santa Cruz Biotechnology (Dallas, TX, USA). Anti-ACTIN (cat# A5316) was from Sigma Aldrich (St. Louis, MO, USA). Anti-PRMT1 (cat# A300722A) was from Bethyl Laboratories (Montgomery, TX, USA). Anti-TIAR (cat# 610352) and anti-G3BP (cat# 61126) were from BD Biosciences (San Jose, CA, USA).

### Plasmids and reagents

GFP-TDRD3 and its truncated constructs have been described before [13]. GST-Tudor (E691K) was generated by introducing a Glu (E)-to-Lys (K) mutation at amino acid 691 of TDRD3 using site-directed mutagenesis (Agilent Technologies, Santa Clara, CA, USA).

### GST pull-down

GST, GST-Tudor (WT), GST-Tudor (E691K), GST-USP9X (N1), GST-USP9X (N2), GST-USP9X (C1), GST-USP9X (C2) were expressed and purified from *E. coli*. The HeLa cells were lysed in lysis buffer containing 50 mm Tris–HCl, (pH 7.5), 150 mm NaCl, 0.1% Nonidet P-40, 5 mm EDTA, 5 mm EGTA, 1.5 mm MgCl_2_, 5% glycerol and protease inhibitors (Roche, Basel, Switzerland). The purified recombinant proteins were incubated with the HeLa cell lysates overnight at 4 °C. Glutathione Sepharose (GE Healthcare Life Sciences, Pittsburgh, PA, USA) beads were added to the lysate mixture and incubated for 1 h at 4 °C. The eluted samples were loaded on an SDS–PAGE gel and detected by western blotting using the indicated antibodies.

### Immunoprecipitation of arginine-methylated proteins

To detected arginine-methylated proteins, cells were either left untreated or treated with methylation inhibitor AdOx (20 μm) or MS023 (10 μm) for 3–4 days. Cell pellets were lysed in 1× RIPA buffer (20 mm Tris–HCl, (pH 7.5), 150 mm NaCl, 1% NP-40, 0.5% sodium deoxycholate, 0.1% SDS, protease inhibitor) at 4 °C for 1 h. The lysates were sonicated on ice and clarified by centrifugation followed by preclearing with protein G agarose. The lysates were subsequently immunoprecipitated with ADMA and SDMA antibodies. Immunoprecipitated proteins were analyzed by western blots using USP9X antibody. We routinely treated cells with methylation inhibitor for 3–4 days to observe significant methylation inhibition; this is because methylation was considered a rather stable modification.

### *In vivo* ubiquitination

Cells were treated with 10 μm MG132 for 16 h before harvest. Cell pellets were lysed in 1× RIPA buffer (20 mm Tris–HCl, (pH 7.5), 150 mm NaCl, 1% NP-40, 0.5% sodium deoxycholate, 0.1% SDS, protease inhibitor) at 4 °C for 1 h. The lysates were sonicated on ice and clarified by centrifugation followed by preclearing with protein G agarose. The lysates were subsequently immunoprecipitated with the either anti-TDRD3 or anti-MCL-1 antibodies. Immunoprecipitated proteins were analyzed by western blots using indicated antibodies. Anti-p53 blot was used as positive control for proteasome inhibition efficiency. Anti-ACTIN was used as equal loading control.

### *In vitro* methylation

The recombinant proteins of GST-USP9X C2, GST-PRMT1, GST-PRMT3 and GST-CARM1 were purified from bacterial. *In vitro* methylation reactions were carried out in 30 μl of phosphate-buffered saline (pH=7.4.) containing 0.5–1.0 μg of substrate, 3 μg of recombinant enzymes and 0.42 μm S-adenosyl-l-[methyl-3H]methionine (79 Ci/mmol from a 7.5 μm stock solution; PerkinElmer Life Sciences, Boston, MA, USA). The reaction was incubated at 30 °C for 1 h and then separated on SDS–PAGE, transferred to a PVDF membrane, treated with En3Hance (PerkinElmer Life Sciences), and exposed to film for 2 weeks at −80 °C.

### Immunofluorescence

The HeLa cells were grown on glass coverslips to the desired confluence before fixation. The cells were either left untreated or treated with 0.5 mm sodium arsenite for 30 min. The cells were rinsed with PBS and fixed with ice-cold methanol for 20 min at room temperature. After a blocking step with 20% newborn calf serum, the cells were incubated with the indicated antibodies at 4 °C overnight. The cells were then stained with a fluorescence-labeled secondary antibody, and then stained with 4′,6-diamidino-2-phenylindole (DAPI). The coverslips were then sealed and examined using an Olympus BX50 microscope (Toyko, Japan).

### RT-qPCR

The total cellular RNA was extracted by the TRIzol Reagent (Invitrogen, Carlsbad, CA, USA). The RNA was analyzed for integrity using the Agilent 2100 Bioanalyzer (Agilent Technologies). The total RNA (1 μg) was then used as template to synthesize cDNA with the High Capacity cDNA Archive Kit (Applied Biosystems, Foster City, CA, USA) and qPCR was subsequently performed on the CFX96 Real-time System C1000 Touch Thermal Cycler (Bio-Rad, Irvine, CA, USA). The RNA levels were normalized to the endogenous control gene Beta-ACTIN. Data analysis was performed using the Bio-Rad CFX Manager 3.1. The experimental cycle threshold (Ct) was calibrated against the ACTIN control product. All amplifications were performed in triplicate. The DDCt method was used to determine the amount of product relative to that expressed by control sample-derived RNA (onefold, 100%).

### Apoptosis assay

Apoptosis was evaluated using the Annexin V-APC/propidium Iodide (PI) apoptosis detection kit (eBioscience, San Diego, CA, USA). Adherent Tet-on-sh TDRD3-expressing MDA-MB-231 cells were treated with the BCL-2 inhibitors, ABT-263 and ABT-199 (Selleckchem, Houston, TX, USA), and the USP9X inhibitor, WP1130 (Apex Bio, Houston, TX, USA); Camptothecin (Sigma Aldrich) at the indicated doses and desired time points. The cells were collected by trypsinization and stained with Annexin V-APC/PI according to the manufacturer’s instructions. Flow cytometry analysis was performed on a CyAnTM ADP Analyzer (Beckman Coulter, Brea, CA, USA). The percentage positive cells in the lower right (APC positive/PI negative: early apoptotic cells) and upper right (APC positive/PI positive: late apoptotic cells) quadrants were summed to calculate the total number of apoptotic cells.
