# Supplementary material for: Arginine methylation of USP9X promotes its interaction with TDRD3 and its anti-apoptotic activities in breast cancer cells
Source: Cell Discov. 2017 Jan 3;3:16048–. doi: 10.1038/celldisc.2016.48 (PMC5206711; doi:10.1038/celldisc.2016.48)
Supplement: Supplementary Information [file celldisc201648-s1.pdf]

Supplementary Information:

Arginine Methylation of USP9X Promotes its Interaction with TDRD3 and its Antiapoptotic Activities in Breast Cancer Cells

Nithya Narayanan[1], Zhihao Wang[1], Ling Li[2], & Yanzhong Yang[1§]

[1]Department of Cancer Genetics and Epigenetics, Beckman Research Institute, City of Hope Cancer Center, Duarte, CA 91010, USA

[2]Division of Hematopoietic Stem Cell & Leukemia Research, Department of Hematology and HCT, Beckman Research Institute, City of Hope Cancer Center, Duarte, CA 91010, USA

Contains 6 supplementary figures.

Supplementary Figure S1

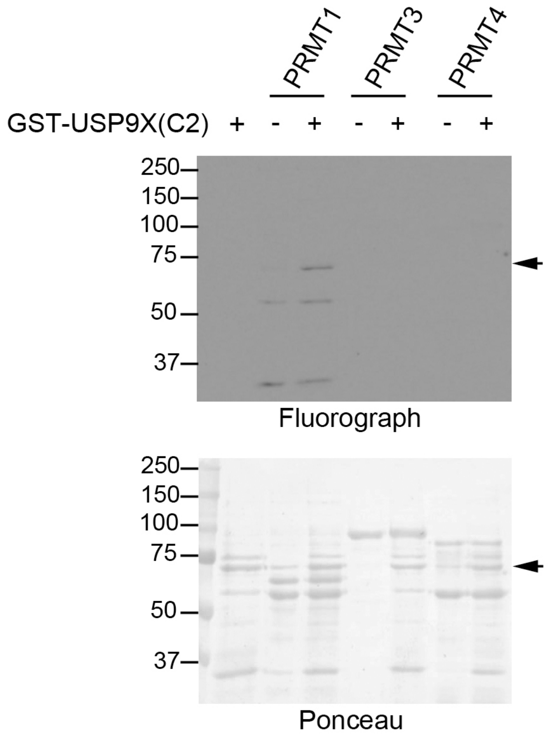

Figure S1. USP9X is methylated by PRMT1 in vitro.

The recombinant GST-UXP9X C2 fragment was incubated with PRMT1, PRMT3 and PRMT4 in the in vitro methylation reaction for 1 hour at 30 °C. The reaction samples were run on a SDS-PAGE gel and detected by radioautography. The membrane was stained with Ponceau to visualize the proteins.

Supplementary Figure S2

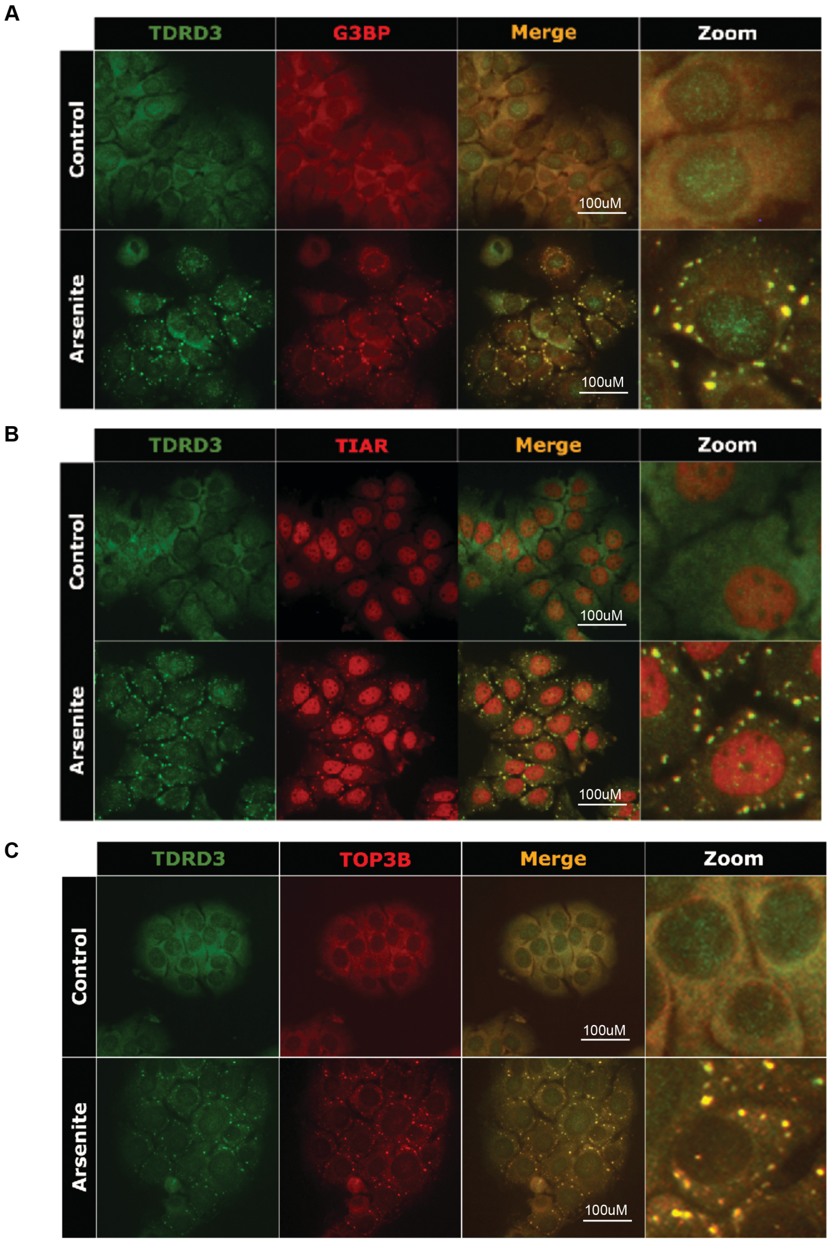

Figure S2. TDRD3 co-localizes with G3BP, TIAR, and TOP3B in cytoplasmic SGs.

(A) HeLa cells cultured on glass cover slips were left untreated (Control) or treated with 0.5 mM sodium arsenite (Arsenite) for 30 minutes. The cells were fixed and immunostained with anti-TDRD3 and anti-G3BP to detect the localization of endogenous proteins. DAPI was used to detect the nuclear DNA.

(B) HeLa cells were treated as in **(A)** and immunostained with anti-TDRD3 and anti-TIAR.

(C) HeLa cells were treated as in **(A)** and immunostained with anti-TDRD3 and anti-TOP3B.

Supplementary Figure S3

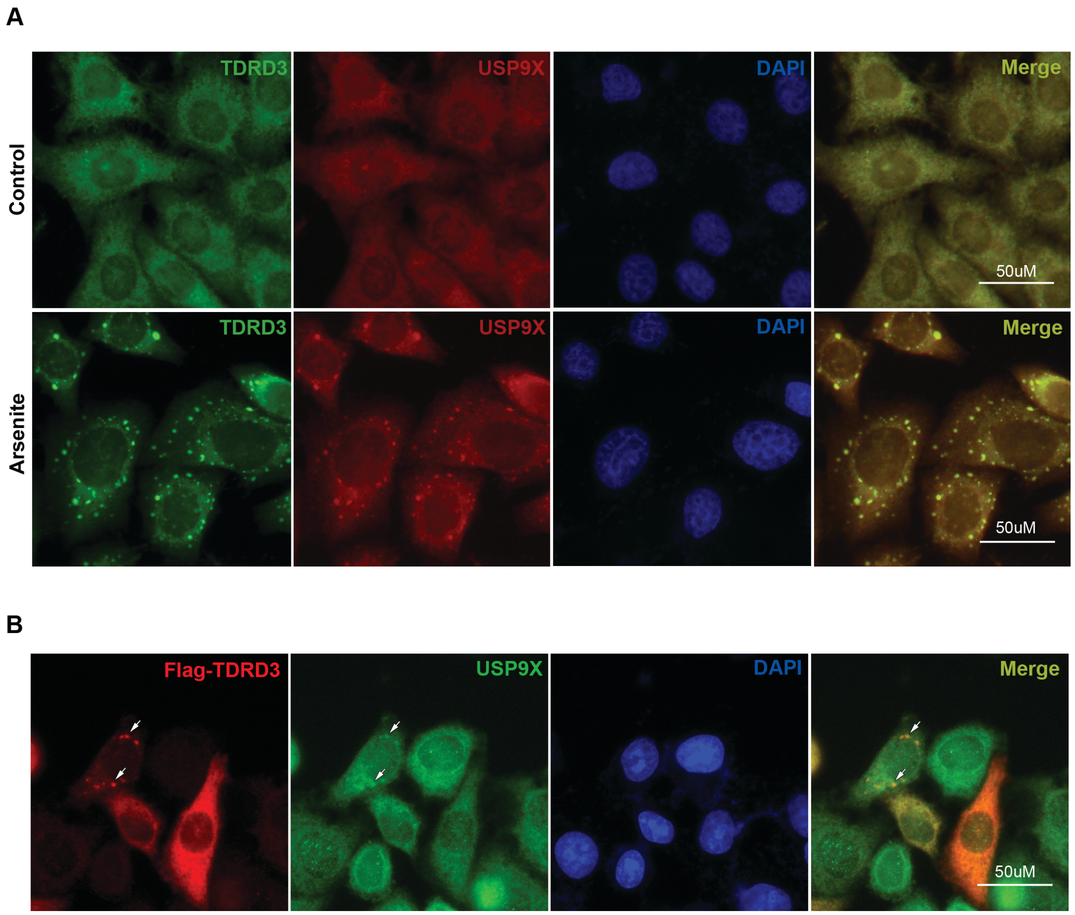

Figure S3. TDRD3 and USP9X co-localize in cytoplasmic SGs.

(A) HeLa cells cultured on glass cover slips were left untreated (Control) or treated with 0.5 mM sodium arsenite (Arsenite) for 30 minutes. The cells were fixed and immunostained with anti-USP9X (ab56461) and anti-TDRD3 to detect the localization of endogenous proteins. DAPI was used to detect the nuclear DNA.

(B) HeLa cells cultured on glass cover slips were transfected with FLAG-TDRD3 for 24 hours. The cells were fixed and immunostained with anti-FLAG and anti-USP9X antibodies to detect the localization of FLAG-TDRD3 and endogenous USP9X. DAPI was used to detect the nuclear DNA. The arrows indicate USP9X and FLAG-TDRD3 co-localization in SGs.

Supplementary Figure S4

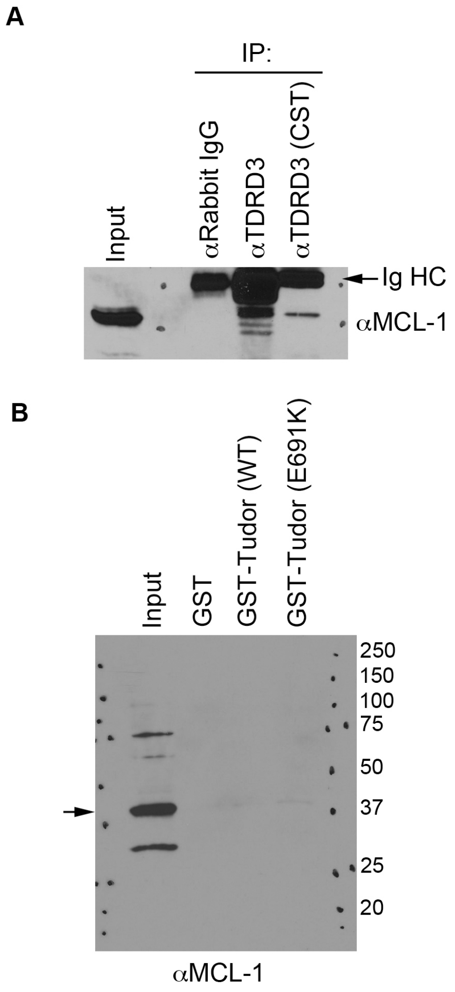

Figure S4. TDRD3 interacts with MCL-1.

(A) TDRD3 co-IPs with MCL-1. Co-IP was performed as described in **Figure 1B**, and the IPed protein complex was detected by western blotting using anti-MCL-1. The arrow indicates the antibody heavy chain.

(B) The Tudor domain of TDRD3 does not interact with MCL-1. GST pull-down was performed as described in Figure 1A, and the pull-down samples were detected by western blotting using anti-MCL-1. The arrow indicates the MCL-1 protein.

Supplementary Figure S5

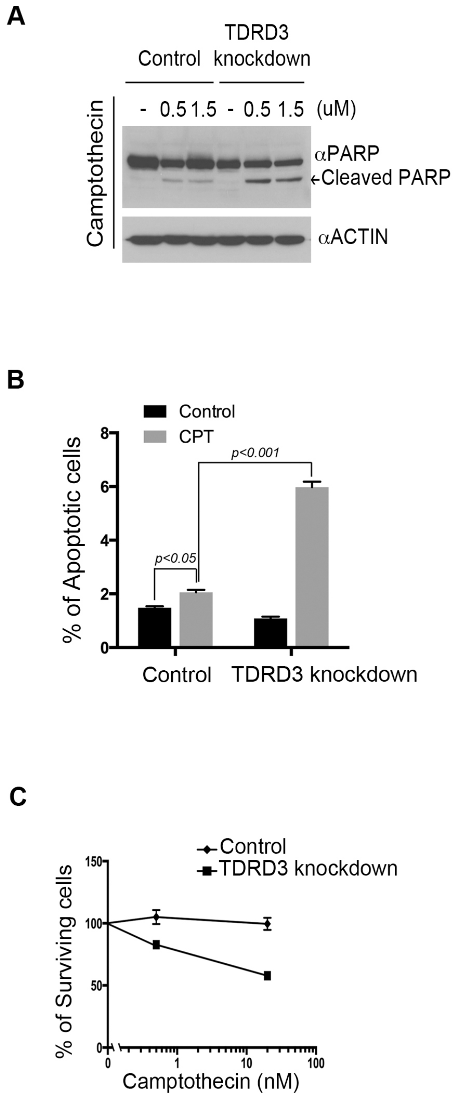

Figure S5. TDRD3 knockdown sensitizes MDA-MB 231 cells to CPT-induced apoptosis.

(A) Both control and Dox-inducible TDRD3 shRNA-expressing MDA-MB-231 cells were treated with increasing amounts of CPT for 24 hours. The total cell lysates were detected with anti-PARP by western blotting. Anti-ACTIN was a loading control.

(B) Both control and TDRD3 knockdown MDA-MB 231 cells were treated with 0.5 μM CPT for 24 hours. Apoptosis was measured by FACS analysis.

(C) Both control and TDRD3 knockdown cells were cultured in the presence of 0.5 and 20 nM CPT for 48 hours. The cell viability was measured by cell counting.

Supplementary Figure S6

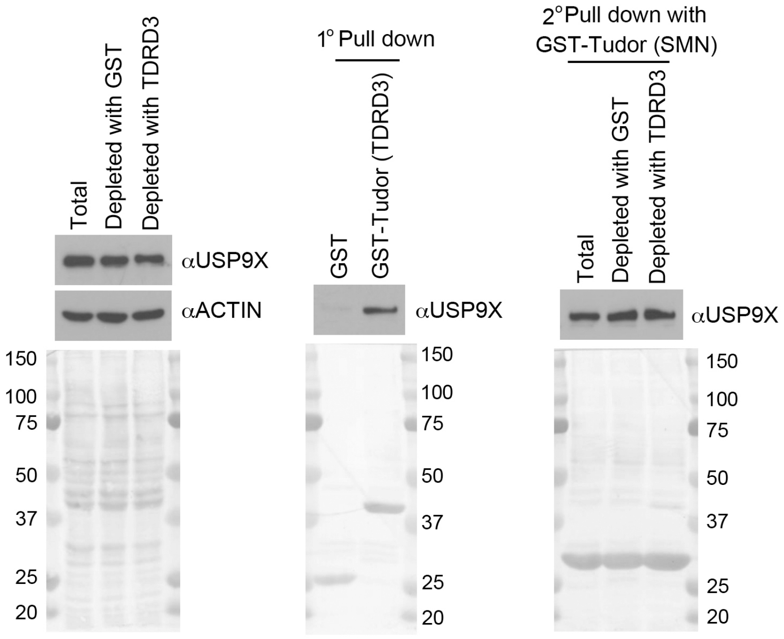

Figure S6. TDRD3 and SMN interact with different pools of USP9X.

GST pull-down experiments were performed using recombinant TDRD3 Tudor domain and SMN Tudor domain. HeLa cell total cell lysates were incubated first with GST or GST-Tudor (TDRD3) to deplete the TDRD3-interacting pool of USP9X (1^0 pull-down). The corresponding supernatants from after GST or GST-Tudor (TDRD3) pull-down, together with equal amounts of fresh HeLa cell lysate, were incubated with GST-Tudor domain of SMN protein (2^0 pull-down). The lysates and the pull-down samples were blotted with anti-USP9X. After western blotting, the PVDF membranes were stained with Ponceau S to monitor the loading.
